# A Novel Nonhuman Primate Model for Influenza Transmission

**DOI:** 10.1371/journal.pone.0078750

**Published:** 2013-11-14

**Authors:** Louise H. Moncla, Ted M. Ross, Jorge M. Dinis, Jason T. Weinfurter, Tatum D. Mortimer, Nancy Schultz-Darken, Kevin Brunner, Saverio V. Capuano, Carissa Boettcher, Jennifer Post, Michael Johnson, Chalise E. Bloom, Andrea M. Weiler, Thomas C. Friedrich

**Affiliations:** 1 Department of Pathobiological Sciences, University of Wisconsin School of Veterinary Medicine, Madison, Wisconsin, United States of America; 2 Wisconsin National Primate Research Center, Madison, Wisconsin, United States of America; 3 University of Wisconsin Microbiology Doctoral Training Program, Madison, Wisconsin, United States of America; 4 Center for Vaccine Research, Dept. of Microbiology and Molecular Genetics, University of Pittsburgh, Pittsburgh, Pennsylvania, United States of America; 5 Vaccine and Gene Therapy Institute of Florida, Port St. Lucie, Florida, United States of America; Johns Hopkins University - Bloomberg School of Public Health, United States of America

## Abstract

Studies of influenza transmission are necessary to predict the pandemic potential of emerging influenza viruses. Currently, both ferrets and guinea pigs are used in such studies, but these species are distantly related to humans. Nonhuman primates (NHP) share a close phylogenetic relationship with humans and may provide an enhanced means to model the virological and immunological events in influenza virus transmission. Here, for the first time, it was demonstrated that a human influenza virus isolate can productively infect and be transmitted between common marmosets (*Callithrix jacchus*), a New World monkey species. We inoculated four marmosets with the 2009 pandemic virus A/California/07/2009 (H1N1pdm) and housed each together with a naïve cage mate. We collected bronchoalveolar lavage and nasal wash samples from all animals at regular intervals for three weeks post-inoculation to track virus replication and sequence evolution. The unadapted 2009 H1N1pdm virus replicated to high titers in all four index animals by 1 day post-infection. Infected animals seroconverted and presented human-like symptoms including sneezing, nasal discharge, labored breathing, and lung damage. Transmission occurred in one cohabitating pair. Deep sequencing detected relatively few genetic changes in H1N1pdm viruses replicating in any infected animal. Together our data suggest that human H1N1pdm viruses require little adaptation to replicate and cause disease in marmosets, and that these viruses can be transmitted between animals. Marmosets may therefore be a viable model for studying influenza virus transmission.

## Introduction

Studies of influenza virus transmission, pathogenesis and immunity rely on animal models to understand processes that are difficult or impossible to investigate in humans. Mice, ferrets, guinea pigs and rhesus macaques have all been used to study various aspects of human infection with influenza viruses [1]. For transmission studies, ferrets are a favored model system due to their susceptibility to unadapted human influenza strains, their development of human-like symptoms during infection and their susceptibility to respiratory droplet transmission. However, ferrets are evolutionarily distant from humans, and a lack of reagents makes immunological studies difficult [2]. Nonhuman primates (NHP) have closer immunological and physiological resemblances to humans; a NHP model may therefore provide the closest possible model for immunity to, and transmission of, influenza viruses in humans. Although macaque monkeys have been used in studies of influenza pathogenesis and immunity [1], transmission of influenza viruses between macaques has not been documented, and there is currently no NHP model for influenza transmission studies. The common marmoset (*Callithrix jacchus*) is an attractive potential model due to its small size and reduced dosing requirements for potential drug and vaccine studies in comparison to macaques. Marmosets are small New World monkeys native to eastern Brazil that breed well in captivity and are already used as models for other viral pathogens, including hepatitis A virus, GB virus B, measles virus, and several hemorrhagic fever and herpesviruses [3].

An understanding of the factors that determine transmissibility of a virus within the human population is indispensible for making informed predictions about the pandemic potential of emerging influenza viruses. Although influenza virus transmission has been studied in a number of models, several questions remain unanswered. The dose required to start a new infection, an important parameter for predicting transmission rates within the human population, is still unknown. Although a few amino acid substitutions have been linked to transmissibility in various influenza subtypes [4], the kinetics by which transmissible variants arise and persist in a population remain unclear. Additionally, the degree to which natural selection may affect transmission between animal hosts is poorly understood. These facets of transmission merit further study because they inform how and why certain influenza viruses are able to emerge and cause pandemics, while others are not. Animal models that mimic human transmission offer a controlled way to study transmission and answer questions, like these, that are impossible to study in humans. Development of a system that is capable of simultaneously modeling human transmission, pathogenesis and immunity would allow combined studies of all of these aspects of influenza infection in a way that is not currently feasible.

Here we sought to determine whether a human influenza A virus could cause disease in, and be transmitted between, marmosets. A human H1N1pdm isolate, A/California/07/2009 (CA/07/09), replicated to high titers in all inoculated animals by 1 day post-infection and, in one instance, was transmitted between cohoused animals. Infected marmosets showed several human-like symptoms, including sneezing, nasal discharge, labored breathing and evidence of lung damage. Deep sequencing revealed that few, if any, genetic changes were required for efficient replication and transmission of this human influenza virus isolate in marmosets, suggesting that marmosets may be viable models for future influenza virus transmission studies.

## Materials and Methods

### Animal infections

Four pairs of HI-confirmed CA/07/09-naïve common marmosets (*Callithrix jacchus*) from the Wisconsin National Primate Research Center (WNPRC) were co-housed throughout the experiment. One animal in each pair was inoculated with 1×10^8^ plaque-forming units (PFU) of an influenza A/California/07/2009 stock virus grown on Madin-Darby canine kidney (MDCK) cells. A total volume of 0.2 ml of virus stock was used for each animal, with 0.1 ml delivered intratracheally, 0.02 ml inoculated to each tonsil, 0.02 ml to each nostril, and 0.01 ml to each conjunctiva. Bronchoalveolar lavage (BAL) and nasal wash (NW) samples were collected from index animals on days 1, 3, 6, 9, 13 and 22 post-inoculation. BAL and NW samples were taken from the contact animals at the same intervals, but offset by two days such that samples were collected on days 3, 5, 8, 11, 15, and 22 days post-contact. Body temperatures were assessed using rectal thermometers, and were recorded with body weight at each timepoint at which BAL and NW were collected. Symptoms were monitored each day throughout the study. All animals cleared influenza virus infection within 3 weeks of inoculation and were returned to the WNPRC colony.

### Ethics statement

No animals were sacrificed during this study. All animals were cared for according to a protocol approved prior to the start of the study by the University of Wisconsin Graduate School Animal Care and Use Committee according to guidelines set by United States National Research Council and the Weatherall Report [5]. All study animals were anesthetized with 100 mg/ml of ketamine for the initial inoculation and all subsequent bronchoalveolar lavages and nasal washes. All animals were kept on a 12-hour light/dark cycle and the study animals were co-housed in a single room separated from the rest of the colony. Veterinary staff provided daily health checks on all animals twice each day, and at the end of the study, all eight study animals were returned to the colony. Each animal was fed 20 grams of marmoset-specific diet twice daily, and water levels were continually monitored. To provide environmental enrichment, a nest box, wooden perches or branches, a hanging toy, and a ladder were present in each cage, and a foraging device was provided with the afternoon feeding at a minimum of once per week.

### HI assays

Hemagglutination inhibition (HI) assays were used to assess antibody responses to H1N1pdm as previously described [6]. HI assays were performed using turkey red blood cells and A/California/07/2009 before infection and three weeks post-infection. Values indicate reciprocal serum titer at which hemagglutination was no longer observed.

### Cytokine detection

A cytokine detection assay was used to assess T cell responses in the study animals. The luminex assay was performed using a 96-well flat bottom plate and a custom set of magnetic beads designed using antibodies specific for human cytokines and chemokines conjugated to magnetic beads from Bio-Rad (Bio-Rad, Hercules, CA). The conjugated magnetic beads were mixed in a 0.5% bovine serum albumin in 1× PBS and were used to coat a 96-well flat bottom plate. BAL samples collected in 1× PBS were tested undiluted in a volume of 50 µl. A mixture of the conjugated magnetic beads was added to the plate, which was then washed 2 times. Samples and standards were then added to the coated plate and incubated for 30 minutes at room temperature on an orbital shaker at 300 RPM. The plate was then washed 3 times. After the wash, detection antibody was added to the plate and incubated at room temperature for 30 minutes on an orbital shaker at 300 RPM. The plate was then washed 3 times. After the wash, Streptavidin-PE was added to each well and incubated for 10 minutes at room temperature in the dark on an orbital shaker at 300 RPM. The plate was washed 3 times and resuspended in 125 µl assay buffer (provided by Bio-Rad). Using Bio-Rad's Bio-Plex Pro software, each analyte was quantified by comparison to standards provided in the kit.

### Symptom assessment

We developed a scoring system to assess the severity of respiratory tract symptoms in infected marmosets. Animals were observed by veterinary care staff each day of the study and symptoms were recorded. Symptoms were scored as being either present or absent, without subjective judgment regarding varying levels of severity. Our scoring system is as follows: development of nasal discharge, 1 point; sneezing, 1 point; labored breathing, 3 points. Sneezing and nasal discharge are common, minor symptoms observed in human influenza cases [7]. In contrast, labored breathing and other signs of respiratory distress are only usually observed in very severe human influenza infections [8]–[10] and are considered much more severe clinical symptoms for which hospitalization is often required. As such, we assigned more points for the development of labored breathing in infected marmosets. Scores for each day were calculated and compared at the end of the study.

### Lung pathology

Tissue damage in the respiratory tract was assessed by quantifying total protein levels in BAL fluid, a method that has been described previously [11]. Each BAL sample was assessed in triplicate, in three independent experiments, using the Quick Start Bradford Protein Assay (Bio-Rad, Hercules, CA) following the manufacturer's protocol. Sample protein concentrations were calculated by interpolation to a standard curve made up of pre-quantified dilutions of bovine serum albumin (Bio-Rad, Hercules, CA), with optical density measured at 600 nm. Both the microassay and standard protocols were used, depending on the total concentration of protein in each sample. Samples that had to be diluted for the microassay protocol were diluted in water. Total protein levels in the BAL fluid of infected and uninfected animals were compared using an unpaired t-test with Welch's correction in Prism version 6.0b for Mac (GraphPad Software, La Jolla, CA, www.graphpad.com).

### Measuring influenza virus replication

Viral replication was monitored by standard plaque assays performed in duplicate on MDCK cells and by QRT-PCR using a Taqman assay with the primers F: 5′-GGACTGCAGCGTAGACGCTTT-3′, R: 5′-3′CATCCTGTTGTATATGAGKCCCAT and the probe 5′-6-fam- CTHAGYTATTCWRCTGGTGCACTTGCC-BHQ1-3′. Viral RNA was isolated using the Maxwell System (Promega, Madison WI) for Total Viral Nucleic Acid. Viral RNA was reverse transcribed and amplified using the Superscript III One-Step RT-PCR System (Invitrogen, Grand Island, NY) and quantified using a LightCycler 480 (Roche, Indianapolis, IN). Cycling conditions were as follows; 37°C for 15 min, 50°C for 30 min, 95°C for 2 min, then 50 cycles of 95°C for 15 sec and 60°C for 1 min. Serial dilutions of a synthetic transcript of the influenza M gene were used to prepare an internal standard curve in each assay. The limit of detection for the assay is 100 copies/ml.

### Sequencing influenza virus genomes

Viral RNA was isolated from BAL and NW samples using the RNeasy Mini Kit (Qiagen, Germantown, MD). Single-stranded DNA was generated with the Superscript first-strand synthesis kit (Invitrogen, Grand Island, NY) using the UniM primer [12] bound to the 5′ and 3′ terminal repeat regions and following the manufacturer's protocol. PCR amplification was performed using the Phusion high-fidelity DNA polymerase kit (New England BioLabs Inc., Ipswich, MA) with segment-specific primers targeting the 5′ and 3′ terminal repeat regions. Primer sequences are listed in **Table S1 in File S1**. Cycling conditions were as follows: 98°C for 30 seconds, followed 35–40 cycles of 98°C for 15 seconds, 62°C for 15 seconds (PB2, PB1, PA, and HA genes, 58°C for NP, NA, M, and NS genes), and 72°C for 1 minute and 20 seconds, followed by a 72°C for 10 minutes and a hold at 10°C.

PCR products were purified from a 1% agarose gel using the QIAquick Gel Extraction Kit (Qiagen, Germantown, MD) following the manufacturer's protocol, and quantified with the Qubit dsDNA HS Assay kit (Invitrogen, Grand Island, NY). For sequencing preparation, DNA was diluted in DEPC-treated water to a concentration of 2 ng/µl. Samples were prepared for sequencing using the Nextera DNA Sample Preparation Kit (Illumina, San Diego, CA) according to the manufacturer's protocol with slight modifications. DNA concentration was re-quantified using the Qubit dsDNA HS Assay kit and average DNA fragment length was determined using the Agilent High Sensitivity DNA kit and the Agilent 2100 Bioanalyzer (Agilent, Santa Clara, CA). Prepared samples were pooled together to a total concentration of 2 nM and prepared for the Illumina MiSeq following the Illumina Sample Preparation Guide. All samples were loaded as a 6 pM library with 1% PhiX and run with a 300-cycle kit. Output files were generated in fastq format.

### Sequence data analysis

Sequence data was imported into CLC Genomics Workbench Version 6 (CLC Bio, Denmark) for analysis. All reads were trimmed using a quality score threshold of 0.001, which corresponds to a Q30 score. A reference genome was compiled by mapping the sequenced stock virus reads to A/California/07/2009 sequences downloaded from GenBank (accession numbers are listed in **Table S2 in File S1**) and extracting the consensus sequence. The consensus sequence was generated using the majority base at each nucleotide position, with reads required for all regions of the gene in order to generate the consensus. Conflicts were resolved by voting at each base, and ambiguity nucleotides were not used. All sample reads were mapped to these consensus reference sequences. SNPs were called in CLC Genomics Workbench, Version 5.5.2 (CLC Bio, Denmark) using a frequency threshold of 1%, requiring regions to have a minimum coverage of 100 reads and a central base quality score of Q30 or higher.

### Computational Methods

The π statistic for measuring nucleotide diversity was calculated in PoPoolation version 1.2.2 [13] using the Variance-at-position.pl script. A minimum coverage of 100 was required for each sequence.

## Results

### A human H1N1pdm influenza virus isolate replicates to high titer and is transmitted between marmosets

**Figure 1:**
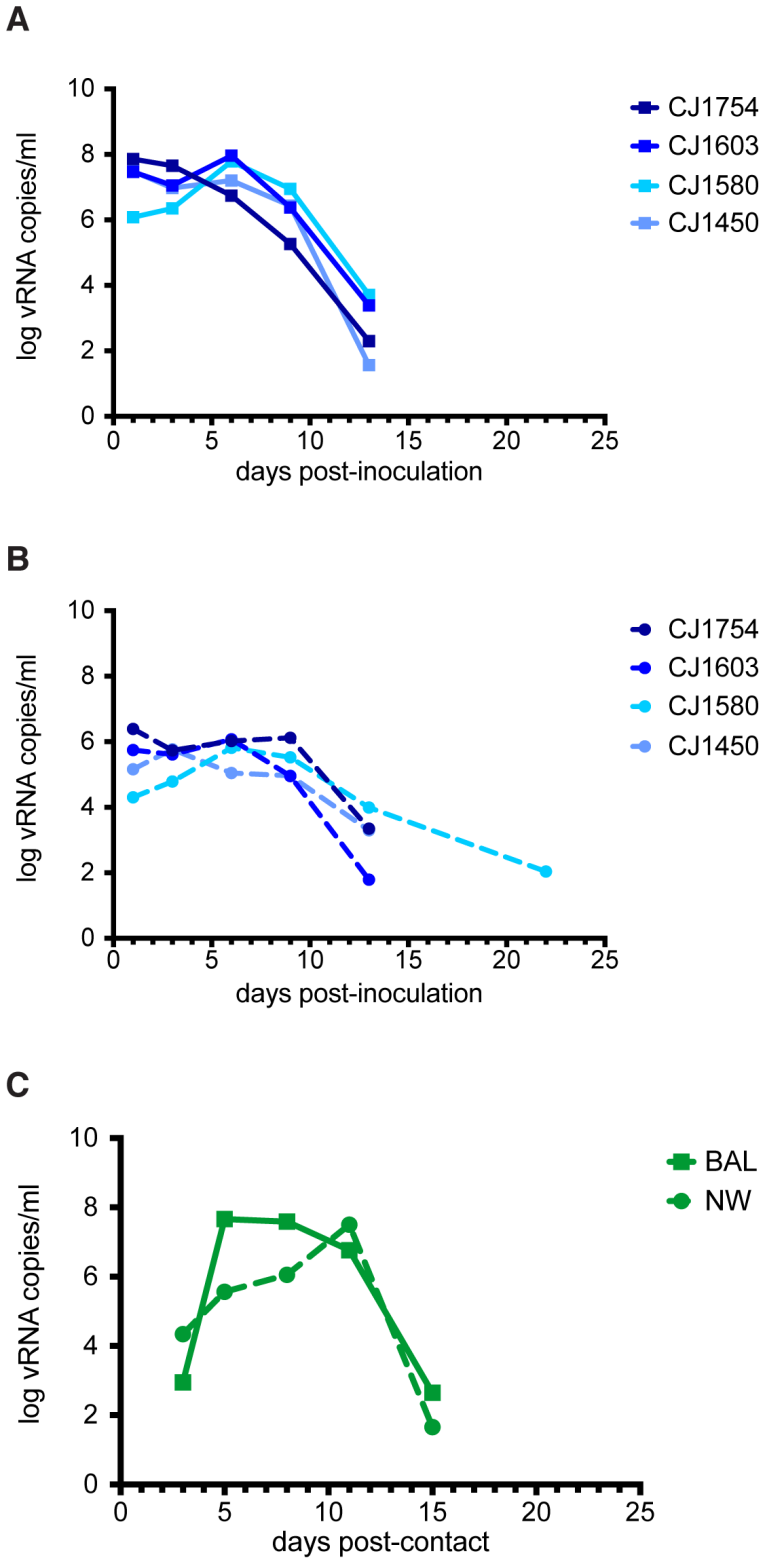
The human influenza virus isolate A/California/07/2009 replicates to high titers in common marmosets. Log vRNA copy number was determined by QRT-PCR in (A) the lower respiratory tract using bronchoalveolar lavage (BAL) and (B) in the upper respiratory tract using nasal washes (NW). (C) Viral RNA was also detected in the lower (solid traces) and upper (dashed traces) respiratory tracts of one contact animal, CJ1721.

At the outset of the study, we first screened animals for pre-existing antibodies capable of recognizing H1N1pdm using hemagglutination inhibition assays. All animals lacked detectable antibodies capable of neutralizing the human H1N1pdm isolate A/California/07/2009 (CA/07/09) before inoculation (**Table 1**). We next inoculated one animal in each of four cohoused pairs with 1×10^8^ plaque-forming units (PFU) of CA/07/09. Bronchoalveolar lavage (BAL) and nasal wash (NW) samples were collected at regular intervals for three weeks post-inoculation, and viral replication was monitored by QRT-PCR and plaque assay. The human isolate CA/07/09 replicated to high titer in the upper and lower respiratory tracts of all index animals (**Figure 1a and 1b**). Viral RNA was detectable in BAL fluid from infected animals between 1 and 15 days post-inoculation, and in NW fluid from 1 to 22 days post-inoculation. Standard plaque assays showed lower titers of infectious virus in both the upper and lower respiratory tracts and shorter shedding time (**Figure S1**). By three weeks post-inoculation, all four index animals had serum antibody titers between 40 and 160 against CA/07/09 (**Table 1**). In addition, one of the contact animals, CJ1721, the cage mate of CJ1450, had seroconverted (**Table 1**). Analysis of the BAL and NW samples from CJ1721 revealed that CA/07/09 had replicated to high titer in this animal as well, persisting from 3 to 15 days post-contact in both the upper and lower respiratory tracts (**Figure 1c**). These data indicate that the human H1N1pdm isolate was transmitted between animals in one of four marmoset pairs.

**Table 1:** Anti-CA/07/09 serum antibody titers.

	Animal	pre-infection	21 days post-infection
		CA/07/09-specific HI	CA/07/09-specific HI
Index	CJ1754	<10	160
	CJ1603	<10	40
	CJ1580	<10	80
	CJ1450*	<10	80
Contact	CJ1721*	<10	160
	CJ1684	<10	<10
	CJ1681	20	<10
	CJ1589	<10	<10

Anti-CA/07/09 serum antibody titers as determined by standard HI assays using turkey red blood cells and A/California/07/2009 virus.

* indicates transmitting pair.

### Marmosets develop human-like symptoms during infection with H1N1pdm virus

**Figure 2:**
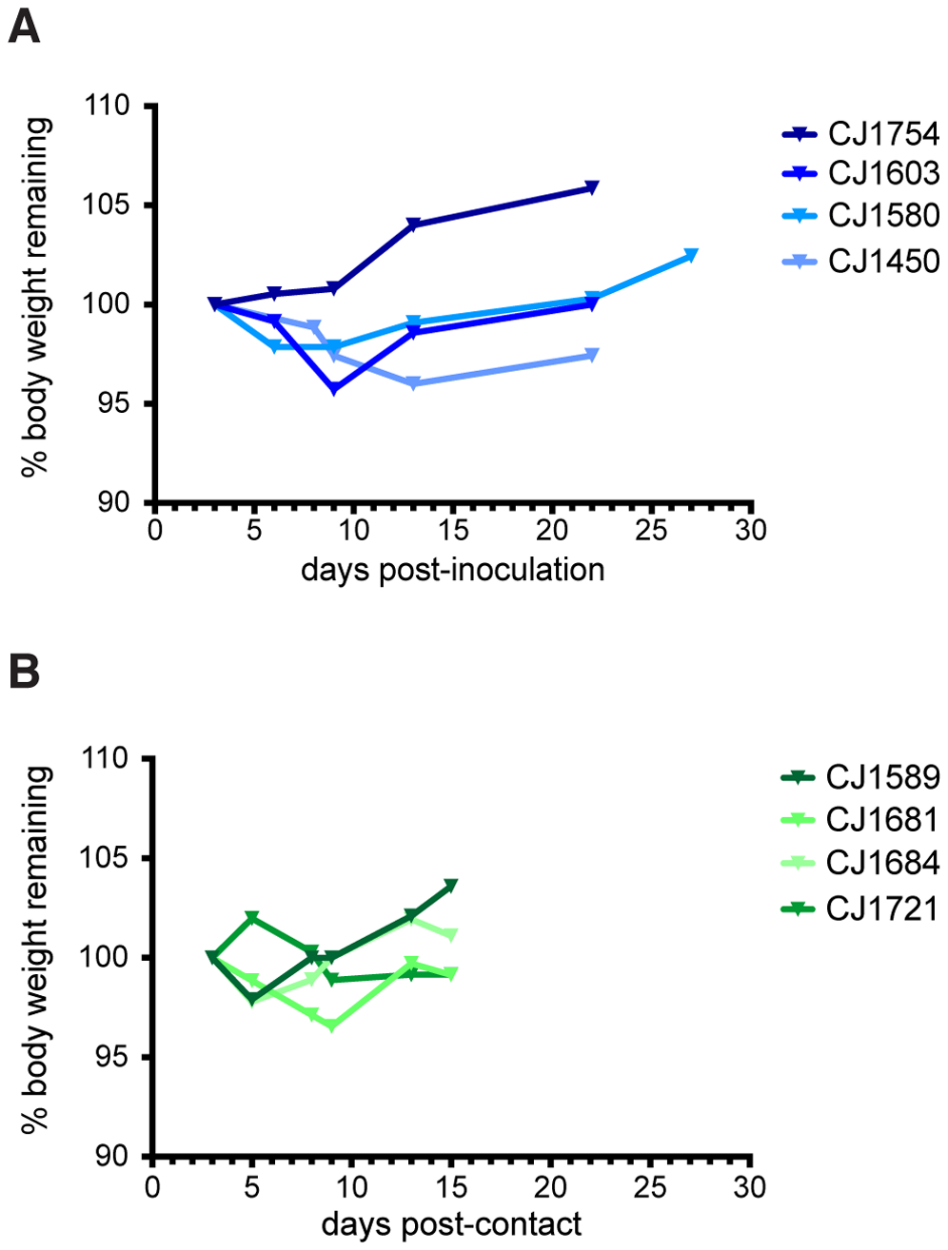
Body weight did not change during the study period in index (A) and contact (B) animals. No statistically significant difference in body weight lost was observed between infected and uninfected animals (unpaired t-test with Welch's correction, p = 0.8355).

**Figure 3:**
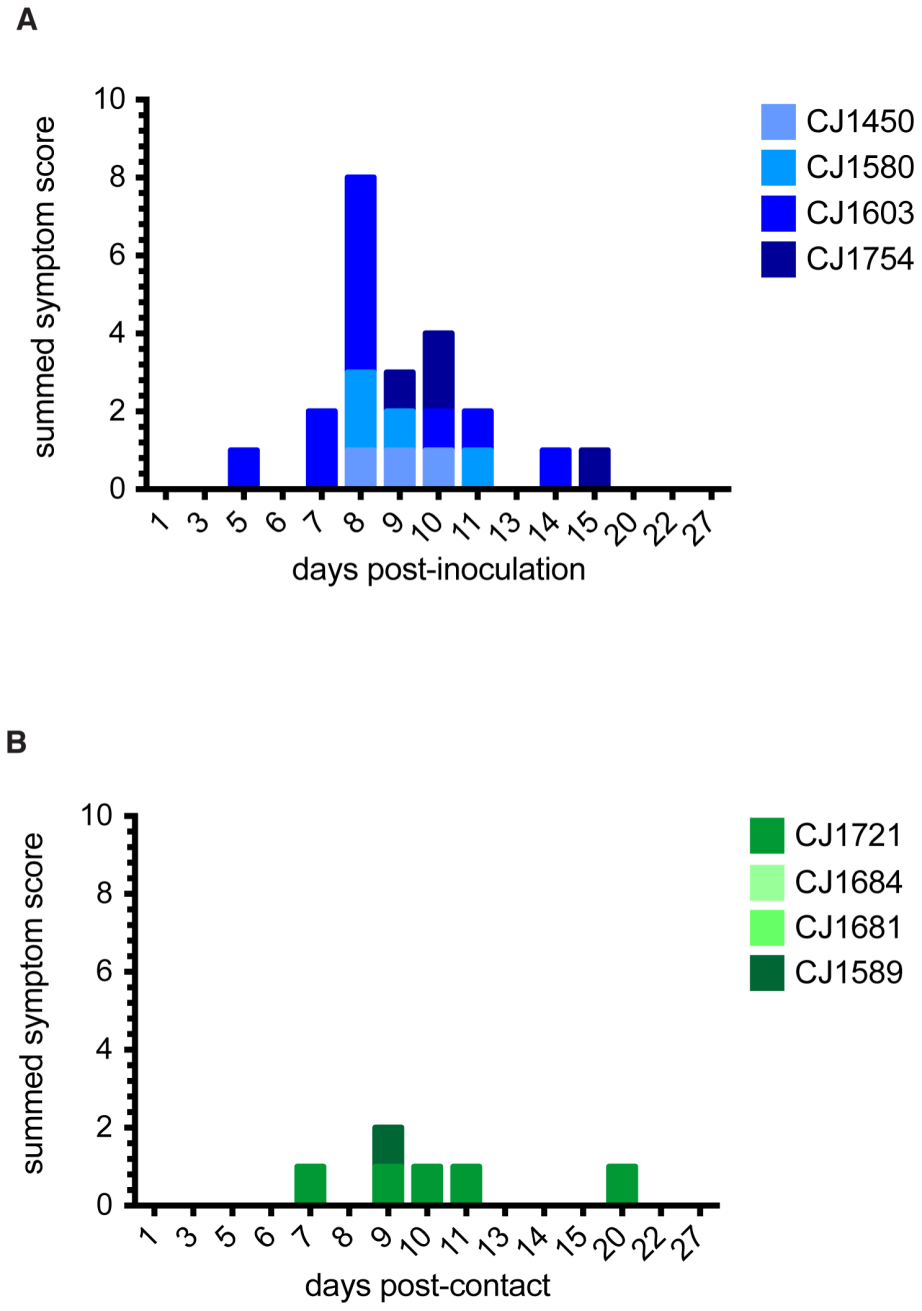
Symptom scores of infected animals reflect development of human-like symptoms. Symptoms were scored as follows: nasal discharge, 1 point; sneezing, 1 point; labored breathing, 3 points. Each animal was observed at least once daily throughout the experiment; symptoms were recorded for the days shown. The graphs show the summed symptom scores for each index (A) and contact (B) animal on each day on which symptoms were recorded.

To monitor symptoms throughout the study, we developed a scoring system to assess symptom onset and disease severity (see Materials and Methods). Weight and body temperature were recorded on days when BAL and NW samples were taken, and symptoms were recorded daily. There was no weight loss (**Figure 2**) or fever (**Figure S2**) associated with infection in any animal. Interestingly, we did observe several symptoms commonly associated with human influenza infection. All index animals developed nasal discharge and sneezing (**Table S3 in File S1**). CJ1603 showed the most severe symptoms, experiencing sneezing, nasal discharge, and on one day, labored breathing. Symptom scores peaked between 8 and 10 days post-inoculation in all index animals, ranging from a score of 2 to a score of 5 (**Figure 3a**). One contact animal, CJ1721, also developed sneezing and nasal discharge on multiple days of the study, and had a single symptom on days 7, 9, 11, and 20 (**Figure 3b**). We also noted a single instance of sneezing in CJ1589, although viral RNA was undetectable in BAL and NW fluid from this animal, and it failed to seroconvert. No symptoms developed in the other two contact animals. The median summed symptom score of infected animals (index animals and CJ1721) was 4 (range = 3–11); in contrast, the median summed score for uninfected animals was 0 (range = 0–1).

### Infection with H1N1pdm virus is associated with lung tissue damage

**Figure 4:**
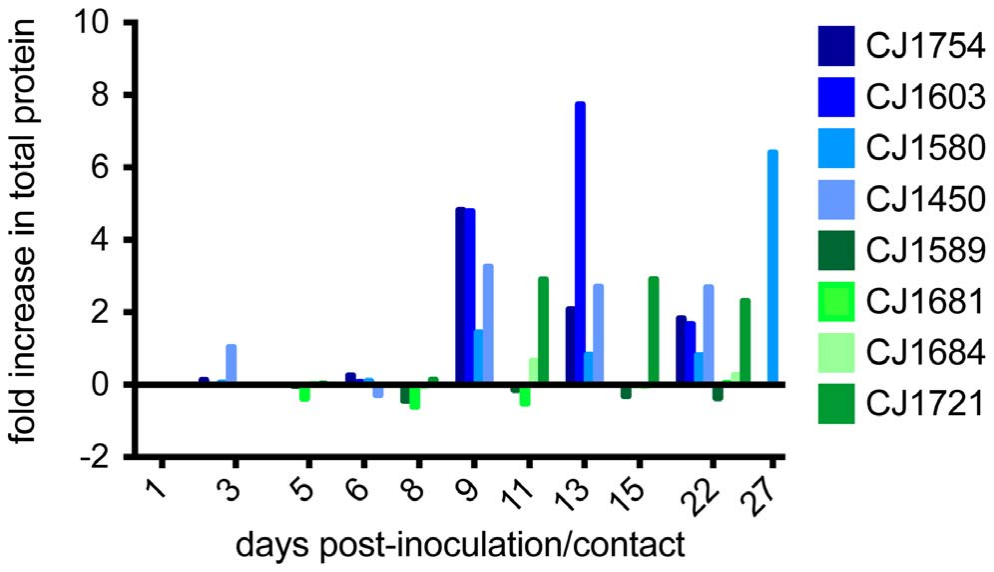
Protein is secreted into BAL fluid as influenza infection progresses. Bradford assay was used to detect secretion of proteins into the lungs as an indicator of damage to lung tissue. Values were normalized to protein levels detected on the first day BALs were collected. Data on subsequent days are expressed as fold increase over this baseline level; therefore negative values on this scale indicate decreased protein concentration in BAL with respect to baseline. Blue bars represent index animals; green bars represent contact animals.

Respiratory tract infection can result in disruption of the alveolar-capillary barrier, edema and leakage of serum proteins into the lungs [11]. Cell lysis will also cause elevated protein levels in the lungs as cytosolic proteins will be released into the extracellular space [11]. To assess lung tissue damage in our study, we quantified total protein present in BAL fluid. All index animals had elevated protein levels in their BAL fluid as infection progressed and all showed a significant increase above day 1 levels by 9 days post-inoculation (**Figure 4**). CJ1754 showed a 4.82-fold increase in BAL fluid protein between days 1 and 9. In this same period CJ1603 showed a 4.79-fold increase, CJ1580 a 1.44-fold increase, and CJ1450 a 3.26-fold increase. CJ1603, the animal with the highest overall symptom score, developed even higher protein levels by day 13, with a 7.73-fold increase in total protein over day 1 levels. CJ1580 showed a delay in peak protein increase, peaking at 27 days post-inoculation with a 6.41-fold increase. BAL fluid from the infected contact animal, CJ1721, also showed an increase in total protein throughout infection, with levels peaking on day 15 (2.91-fold increase over day 1 level). In contrast, the other three contact animals all showed only minor fluctuations in total protein levels throughout the study (range = −0.60–0.66-fold increase). All infected animals had a peak in BAL total protein between days 9 and 27 post-inoculation/contact and an average total protein level of 0.26 mg/ml (range = 0.05–1.03 mg/ml, standard deviation = 0.23); uninfected animals had an average BAL protein level of 0.089 mg/ml (range = 0.052–0.14 mg/ml, standard deviation = 0.03; p = 0.0002, **Figure S3**).

### Cytokines are detectable in NW and BAL fluid

To assess immune responses to influenza infection in the marmoset lung, we used a commercially available assay to detect cytokines present in BAL fluid throughout infection. Reagents validated for rhesus macaques were used to measure levels of interleukin (IL)-17; IL-1β; IL-9; monocyte chemotattractant protein 1 (MCP-1; also known as CCL2); macrophage inflammatory protein 1β (MIP-1β; also known as CCL4); and regulated upon activation, normal T cell expressed and secreted (RANTES; also known as CCL5) in the lungs of all index and contact marmosets. Although poor cross-reactivity was observed for IL-17, IL-1β, IL-9 and MCP-1, reagents for detection of RANTES and MIP-1β in rhesus macaques cross-reacted with marmoset chemokines. Levels of both of these molecules peaked around day 10 in the lungs of all infected animals (**Figure S4**), consistent with the peak in frequency of activated T cells observed in the lungs of influenza-naïve macaques [14].

### Low levels of within-host diversity are maintained during H1N1pdm replication in marmosets

Although the human CA/07/09 isolate replicated to high titer and caused symptoms in marmosets, the fact that transmission occurred in only one pair suggested that this human virus might not have been optimally adapted for replication and/or transmission in these animals. We therefore used deep sequencing to characterize the entire genomes of viruses replicating in each animal to determine whether there was evidence for adaptation of CA/07/09 to marmosets. As a measure of overall within-host viral genetic diversity, we calculated the average nucleotide diversity (π) across each individual gene segment at every timepoint during the study. The π statistic is commonly used to quantify population level diversity [15]; here, it quantifies the average level of heterogeneity at each site in the sequence and is expressed as the average number of substitutions per nucleotide site. Since influenza viruses have high mutation rates, they exist in infected hosts as “swarms” of related, but distinct, sequences. The stock virus used in this experiment was itself a multiply passaged biological isolate, i.e., not produced using reverse genetics, so we expected that the stock would contain some level of genetic heterogeneity. We therefore first used π to quantify the level of diversity present in the stock. The stock virus was characterized by low levels of diversity, with π values ranging from 0.00067 to 0.0016 substitutions per site (**Figure S5**). π could not be calculated for M1 or M2, due to low coverage on parts of the terminal ends of the sequences.

If CA/07/09 had required significant adaptation to replicate in the marmoset host, natural selection could be expected to promote changes in overall diversity. An increase in average nucleotide diversity in a gene segment would suggest that replication in the new host favors a diversifying swarm of viruses, while a decrease in average diversity could suggest that selection is favoring a subset of fit genotypes. Viral replication within the marmoset host did not appear to greatly alter levels of within-host diversity within the first days after infection. Estimated π values among all gene segments in index animals were very similar to levels observed in the stock virus, ranging from 0.00027 to 0.0028. Similar values were observed across the genomes of viruses replicating in the index animals throughout the remainder of infection (**Figure S5**).

We next sought to determine whether transmission resulted in major changes in the genetic diversity of the viral swarm by calculating π for each gene segment in the index and contact animals at the timepoints surrounding transmission. We observed limited changes in overall nucleotide diversity in the PB1, PA, NA, and NS genes during transmission (**Figure S5**), indicating that transmission did not significantly alter the intra-host diversity of these segments. However, we did observe slightly higher levels of diversity in the PB2 and M1 genes from the BAL samples, and in both the BAL and NW samples in the HA and M2 genes (**Figure S5**). Following the first timepoint after transmission, diversity in the contact animal immediately returned to levels similar to those observed in the index animals. Overall, these data suggest that although a subset of genes experienced a transient increase in diversity in the contact animal immediately following transmission, replication within and transmission between marmoset hosts did not significantly alter diversity of CA/07/09.

### Little adaptation is required for H1N1pdm replication in marmosets

**Figure 5:**
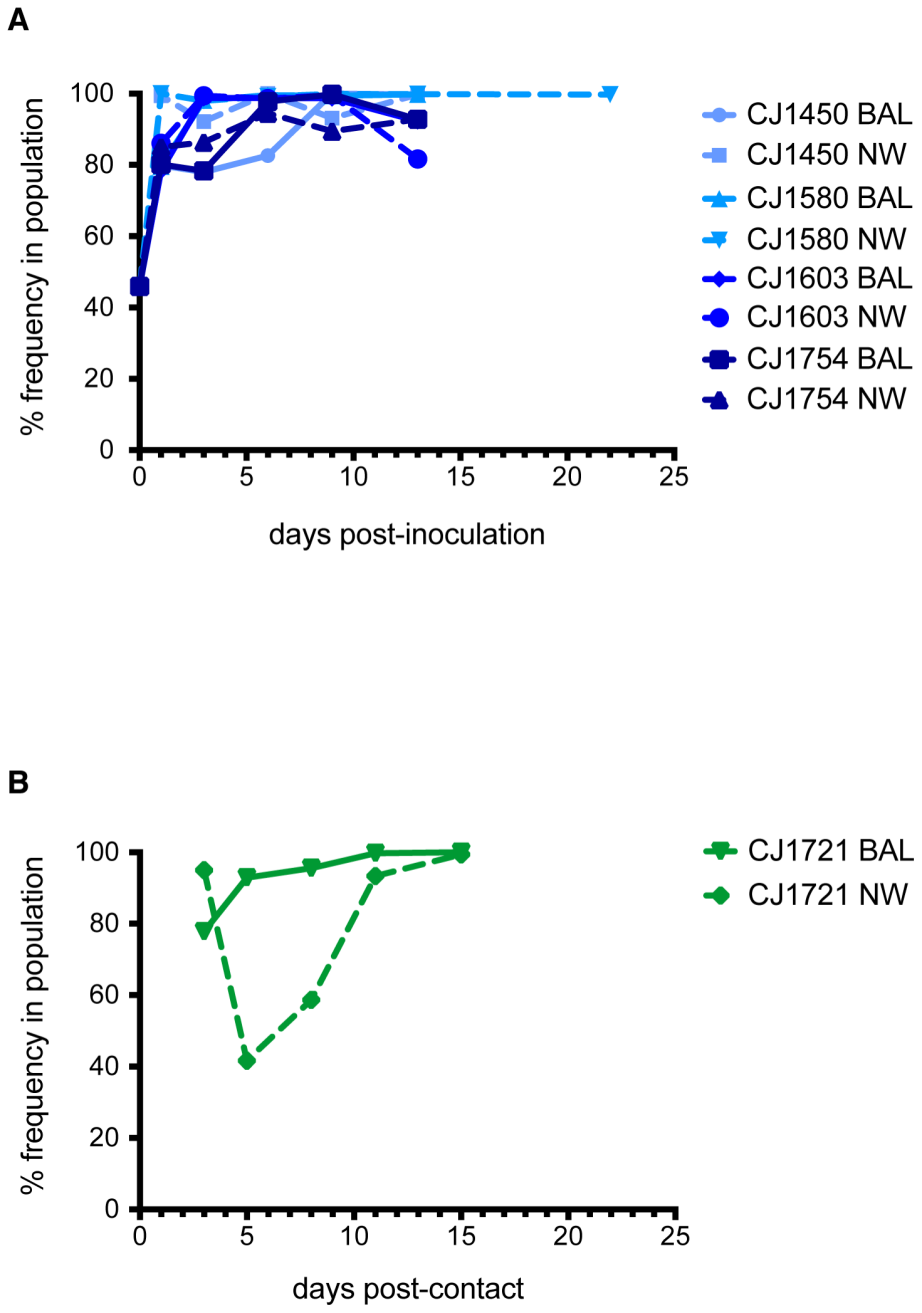
The NP D53E mutation is rapidly fixed in all infected animals. (A) D53E quickly rises in frequency in all index animals. (B) D53E is transmitted to CJ1721. Frequencies in the upper and lower respiratory tracts in CJ1721 right after transmission closely resemble frequencies in the index animal CJ1450 at the time of transmission.

If CA/07/09 required extensive adaptation to replicate efficiently in marmosets, one would expect adapting mutations, whether present in the stock virus or arising de novo, to increase in frequency in the viral population over time. Analysis of single nucleotide polymorphism (SNP) frequencies throughout the viral genome and throughout the course of infection revealed the fixation of only one nonsynonymous substitution that was common to all infected animals, which encodes an aspartate-to-glutamate change at amino acid position 53 in the NP protein (NP D53E). This SNP was present in the stock virus at a frequency of 46% and rapidly rose to fixation in all index animals (**Figure 5a**). Similarly, viruses replicating in the contact animal CJ1721 shortly after transmission had a high frequency of mutations encoding NP D53E (**Figure 5b**). Interestingly, there was a transient decrease in the frequency of this SNP after transmission in CJ1721's upper respiratory tract, but not in the lower respiratory tract. By day 11 post-infection, more than 90% of viruses in both the upper and lower respiratory tracts of CJ1721 encoded NP D53E (**Figure 5b**).

No other nonsynonymous mutations with respect to the CA/07/09 consensus were maintained at a high frequency in every animal. These data indicate that, with the exception of the mutation encoding NP D53E, there were no mutations in CA/07/09 that were consistently selected during viral replication in marmosets. Taken together with low levels of intra-host diversity maintained throughout replication in marmosets, our data suggest that H1N1pdm isolated from humans can replicate in marmosets with little adaptation.

### A mixed population of high- and low-frequency variants is transmitted

**Figure 6:**
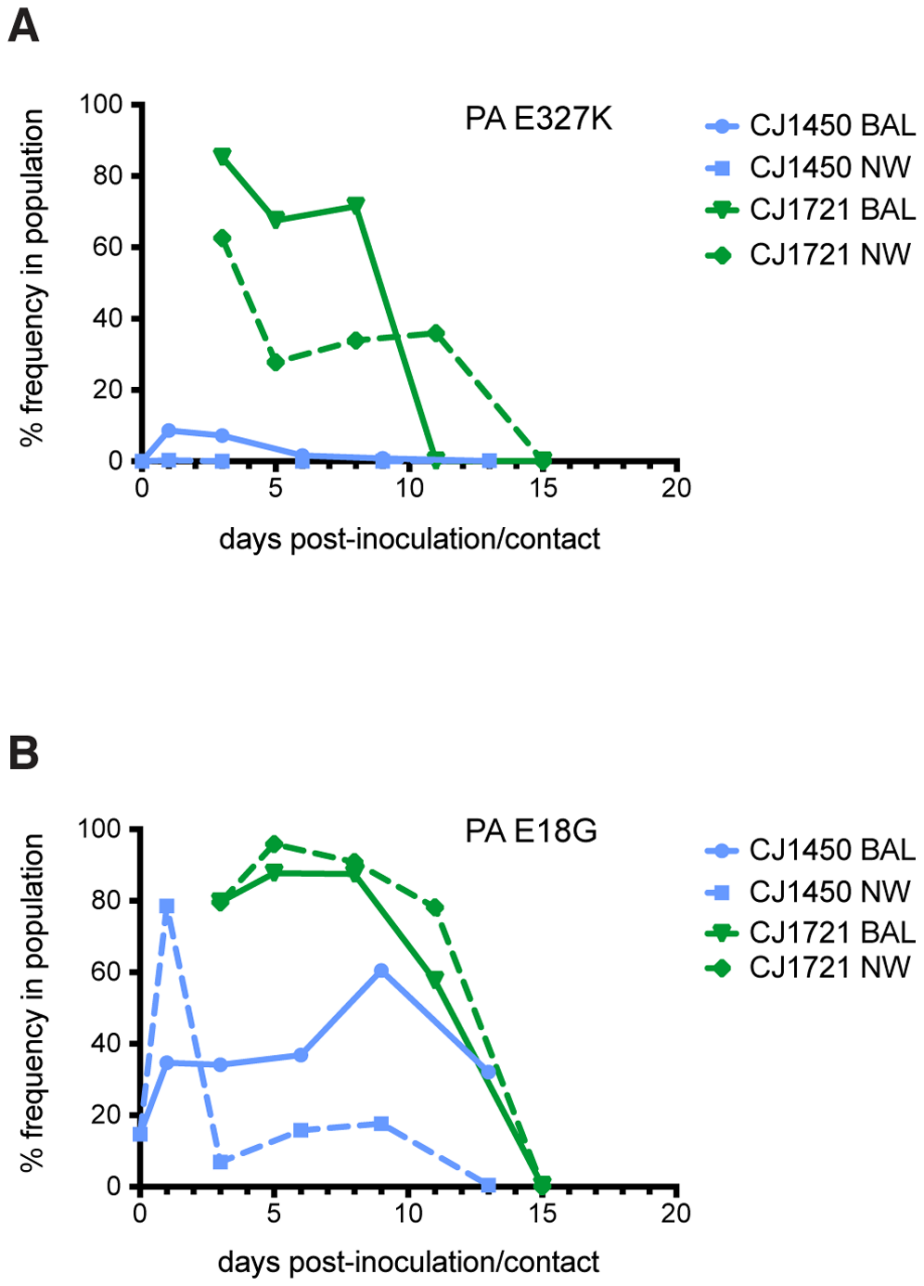
Variants present at both low and high frequencies are transmissible. (A) PA E327K was present in less than 10% of the viruses replicating in the index animal but was present at a high frequency in both the lung and nasal secretions of CJ1721 shortly after transmission. This variant frequency declined in CJ1721 after transmission. (B) PA E18G was present at a frequency around 80% in CJ1721 directly after transmission, but declined over the remainder of the study.

Although only one nonsynonymous mutation consistently became fixed during replication of CA/07/09 in marmosets, we next wanted to determine whether transmission altered the frequency of other individual SNPs, to assess possible selection of variants during transmission. We therefore analyzed SNPs present in CJ1721's virus at the first timepoint after transmission (3 days post-contact). We observed a number of nonsynonymous SNPs present at low to intermediate frequencies (1%–40%) as well as three SNPs present at the consensus level (greater than 50%) in viruses isolated from the BAL and NW of CJ1721 on the first day after transmission. Because our aim was to assess any strong signatures of selection on the viral genome during transmission, we focused our analyses on these three mutations. First we found the mutation encoding NP D53E, which became fixed in all animals within 1–11 days after infection, as discussed above. This substitution was present in CJ1721 at a frequency of 96% in the upper respiratory tract and 76% in the lower respiratory tract, and was maintained at a high frequency for the remainder of infection (**Figure 5b**). These frequencies very closely mimic the frequencies observed in CJ1721's cage mate CJ1450 on day 1 post-inoculation, in which NP D53E was present at a frequency of 99% in the NW and 80% in the BAL (**Figure 6a**).

The second SNP we observed in viruses isolated from CJ1721 resulted in a glutamate-to-lysine change in the PA gene at position 327 (PA E327K). This SNP was present in CJ1721 at a frequency of 62% in the upper respiratory tract and 86% in the lung, although its frequency declined with time and it was lost by day 13 (**Figure 6a** **, green traces**). Interestingly, this particular mutation was only present at a low frequency in CJ1721's cage mate, CJ1450. On both days 1 and 3 post-contact, PA E327K was present at a frequency of 8% in the lung of CJ1450 and was undetectable in its upper respiratory tract (**Figure 6a** **, blue traces**). Therefore, it appears that this transmitted viral variant comprised only a small percentage of the viruses present in the index animal population.

We also observed a second SNP in the PA gene of viruses infecting CJ1721, resulting in a glutamate-to-glycine substitution at amino acid position 18 (PA E18G). This mutation was present at a frequency of 80% in both the upper and lower respiratory tracts of CJ1721 on day 3 post-contact (**Figure 6b** **, green traces**) and at frequencies of 35% in the lung and 79% in the nasal secretions of CJ1450 (**Figure 6b** **, blue traces**). The frequencies of both SNPs observed in the PA gene declined in CJ1721 after transmission and were present at very low levels by the time infection was cleared. This suggests that these amino acid substitutions, although transmissible, may not be advantageous for replication within the marmoset host.

If infection of CJ1721 had been founded by a single virus, or viruses with a single favored sequence, we would expect most mutations to be fixed shortly after transmission. Instead, both the nasal secretions and lungs of this animal harbored viruses containing SNPs present at intermediate and low frequencies, ranging from 1% to 40%. This suggests that the infecting population was comprised of a heterogeneous population of multiple variants rather than a single variant. Taken together with the low levels of intra-host diversity maintained in the index and contact animals at the timepoints surrounding transmission, these data suggest that multiple members of the source animal's viral population were able to start infection in the new host.

## Discussion

The ability to transmit efficiently between human hosts is a major determinant in whether a particular influenza virus can emerge to cause a pandemic. However, the viral and host determinants of such “transmission fitness” remain unclear, hindering our ability to predict the emergence of pandemic viruses. Current experimental models for influenza transmission rely on small mammals that lack developed reagents for immunology studies and are physiologically different from humans. Although NHP models have been developed for studies of pathogenesis and immunity, they have not yet been used to model transmission. Here, we report for the first time that common marmosets are susceptible to the unadapted H1N1pdm virus CA/07/09. The virus replicated to high titer in the upper and lower respiratory tracts of infected animals, and caused development of human-like influenza symptoms after infection, including nasal discharge, labored breathing and lung damage. Infection induces antibodies targeting CA/07/09 that are detectable in standard HI assays. Finally, high viral RNA levels, seroconversion and appearance of symptoms were also observed in a single contact animal, CJ1721, indicating that the human isolate CA/07/09 was transmitted between co-housed marmosets.

Marmosets may make a suitable NHP model for influenza for a number of reasons. Their small size (average size ranges from 400–420 grams) translates to smaller dosing requirements for potential vaccine and drug studies, which may increase their economy over larger monkeys [3]. Additionally, marmosets do not harbor *Macacine herpesvirus 1* (formerly *Cercopithecine herpesvirus 1*; commonly known as herpes B virus), which is endemic in Asian macaques and can cause fatal encephalitis in humans [3]. Although marmosets have previously been used to study other viral diseases [3], their utility for influenza studies has never been assessed.

Importantly, our study shows that marmosets share many aspects of influenza infection that occur in humans. In a typical human infection, virus shedding begins within the first day after inoculation and persists for eight to nine days [7]. Symptoms generally appear one to four days post-exposure (average is two days), and can include fever, myalgia, malaise, sore throat, cough, headache, sneezing, nasal discharge, and in severe cases labored breathing and lung damage [7]. While other NHP species have been used to study influenza immunity and pathology, they frequently do not develop upper respiratory tract symptoms characteristic of human infection with H1N1 or H3N2 viruses [2]. In our study, all infected marmosets developed human-like symptoms between 5 and 8 days post-contact, indicating disease progression similar to that observed in humans. In this study, marmosets began shedding virus by one day post-inoculation, and viral RNA remained detectable for between 13 and 22 days. This represents a longer infection than what is observed in humans and other animal hosts [7], but it is unclear whether this prolonged shedding is a hallmark of marmoset influenza infection or is due to the relatively high inoculum dose used for this study. For comparison, most experimental inoculations of rhesus macaques use between 2 and 9×10^6^ PFU [16]; human volunteer challenge studies have inoculated between 1×10^3^ and 1.7×10^7^ PFU [7], and ferret studies often challenge with 10^6^ PFU [17]. We chose to use a high inoculum dose in the present study to allow us to unambiguously determine whether marmosets can be infected with human influenza viruses. Although we did not determine a minimum infectious dose in this study, we predict that future work will find that a lower dose can be used to achieve a productive infection.

The development of sneezing in marmosets is important. Human influenza can be transmitted in three ways: via direct contact, large droplets, and small aerosols. While the relative importance of each of these modes of transmission in human cases is not yet clear, all are known to occur, and several studies suggest that transmission by large and small droplets is likely to be the most efficient [18]. Sneezing generates a large number of particles that can vary substantially in size [19]; upon expulsion into the air, small particles can rapidly evaporate and shrink, allowing them to float in air currents without settling for long periods of time [18]. This allows small aerosols to travel for longer distances before settling, facilitating long-range transmission and establishment of infection in the lower respiratory tract. Thus, the development of sneezing in marmosets greatly enhances the likelihood that aerosol transmission could occur. The design of our study did not allow us to determine the mode of transmission that occurred between CJ1450 and CJ1721. Animals were co-housed, meaning that they shared food, water, and living space. Co-habitation can facilitate transmission through direct contact [20], but does not exclude large droplet or aerosol transmission. Small aerosols can be generated from breathing alone [21], and large droplets can arise from breathing, coughing, sneezing or speaking and travel distances up to 3 feet [18], [22]. Although transmission in our study occurred before CJ1450 showed any symptom development including sneezing, this does not exclude the possibility that CJ1450 generated aerosols or large droplets through breathing or vocalizations. Therefore, there is no way to definitively determine how transmission occurred. Although all of the marmoset pairs were kept in the same room, it is unlikely that transmission occurred between CJ1721 and any index animal other than CJ1450 because transmission occurred before any index animals developed symptoms. The first instance of sneezing was recorded in CJ1603 5 days post-infection; vRNA was detected in CJ1721 as early as 3 days post-contact. Given the lack of respiratory symptoms that could facilitate transmission before the transmission event, it is likely that CJ1721 acquired H1N1pdm from the marmoset with which it had the most contact, CJ1450. However, the incidence of sneezing later in infection in all infected marmosets suggests that marmosets may be able to model transmission dynamics of all three modes of human transmission.

Secondary attack rate measures the rate of new disease cases arising from contact with an infected person. Human epidemiology studies conducted during and after the 2009 H1N1 pandemic have used confirmed influenza-positive patients to measure secondary attack rates for their household contacts. These estimates range from 13%–31% [23]–[25], indicating that even in humans, inter-host influenza transmission is not 100% efficient. In contrast, the ferret and guinea pig model systems are characterized by much higher rates of 2009pdm transmission between animals, with some studies reporting transmission rates of 100% [26]–[28]. Although transmission of 2009pdm does not appear to be as efficient in marmosets, the rates observed in our study are within the range of estimated human transmission efficiency. Although this study used a small sample size which limited our ability to assess transmission efficiency in a rigorous manner, our observed transmission rate of 25% is nonetheless within the range of H1N1pdm secondary attack rates estimated for the human pandemic. Should future studies uphold this relatively modest transmission efficiency, we recognize that it could represent a drawback to the marmoset transmission model. However, we do not anticipate that marmosets will replace widely used transmission models like guinea pigs and ferrets. Rather, we predict that a marmoset model could play a role in influenza transmission studies for which guinea pigs and ferrets are currently not suited, by providing the ability to study transmission and immunity simultaneously in the same organism.

This study assessed immune responses to influenza infection by assessing antibody production and using a bead array to quantify a panel of cytokines and chemokines in BAL fluid. We detected a peak in levels of the chemokines RANTES and MIP-1β in lungs at 10 days after infection. This timing is consistent with previously documented peaks in the frequency of activated T cells in the lungs of influenza-naïve macaques infected with human influenza viruses [14]. We therefore speculate that activated T cells may have been secreting the majority of RANTES and MIP-1β in the lungs of infected marmosets, but it is possible that T cells were not, or not the only, sources of these chemokines. We observed limited cross-reactivity between a set of commercial reagents for IL-17, IL-1β, IL-9 and MCP-1 and their cognate antigens in marmosets, despite the fact that these reagents detect macaque antigens. These results show that cross-reactivity between available human and/or macaque reagents and marmoset antigens cannot be assumed; rather, each reagent will have to be validated individually. Although validating a large number of reagents was beyond the scope of this study, previous work has validated some additional immunological reagents for use in marmosets [3], [29]–[42]. As increasing numbers of investigators choose to use marmosets for the study of viral pathogens, the number of validated immunological reagents will likely continue to expand.

In our study, we assessed viral replication by both molecular and culture-based methods. While QRT-PCR showed high viral loads in all infected animals, plaque assays showed a much lower infectious viral titer and a significantly decreased shedding time. Although we cannot definitively account for the low titer of infectious virus detected by plaque assays, several observations suggest that active viral replication did indeed occur in our study. In three of the index animals, viral RNA levels increased after the initial inoculation in both the NW and BAL fluid, and in two of the index animals, viral RNA levels did not peak until 6 days post-infection. Viral RNA rapidly increased after contact in animal CJ1721, which was not directly inoculated. Together, these data suggest that detection of viral RNA was the result of virus replication, and not merely residual RNA from the initial inoculum. The clinical upper and lower respiratory tract symptoms present in all infected animals but absent in uninfected contact animals also support productive influenza infection. Sneezing, nasal discharge, labored breathing, and lung damage would not be expected in the absence of replicating virus. Additionally, the simultaneous increase in viral RNA levels and the fixation of NP D53E strongly suggests viral replication. Finally, sequencing reads showed even coverage across the full length of all gene segments, suggesting that there were not large numbers of defective particles in the NW and BAL fluid of infected marmosets.

Ideally mammals used to model human influenza transmission should be susceptible to unadapted human isolates. Our data show that during replication in the marmoset host, low levels of diversity were maintained across the genome in all index animals. During viral replication, the high error rate of the influenza virus polymerase will result in the generation of mutations each time the genome is replicated [43]. Under conditions that present new selection pressures, such as replication in a novel host, positive selection may act to promote genetic diversity. High diversity can allow the viral population to quickly adapt to new environmental conditions by providing a wider range of genotypes for selection to act upon. Conversely, a virus that is already fit for replication in its host species may not require substantial adaptation to cause productive infection in a new individual, and selection may not play an important role in its evolution [44]. Pandemic influenza viruses are able to cause widespread infection because they are able to both replicate in, and transmit efficiently among, human hosts. Marmosets are evolutionarily close to humans, and a human pandemic isolate that has already achieved high replicative fitness in humans may be able to readily infect and transmit in a closely related host. In our data, the maintenance of low levels of intra-host diversity in the infecting viral swarm and the fixation of only a single, conservative amino acid substitution support this interpretation. Together, these data suggest that the human isolate CA/07/09 was likely already well adapted to replicate efficiently in the marmoset host and required only minimal evolution to cause productive infection.

Animal models can be used to study the evolution of transmitted viral variants, which is important for understanding the molecular determinants of transmissibility. During transmission, a bottleneck in the viral population size can alter the diversity observed in the new host [45]. In our study, transmission did not result in significant changes in the overall nucleotide diversity of any gene segments in the viral swarm, indicating that the viral population did not undergo a strong population bottleneck after transmission to the new host. The presence of several SNPs at intermediate frequencies (between 1% and 40%), as opposed to a few fixed SNPs, right after transmission in CJ1721 suggests that infection was founded by a mixed population of viruses. Interestingly, our data revealed three nucleotide substitutions present at a high frequency in CJ1721 after transmission. While NP D53E was present at strikingly similar levels in the index and contact animals, PA E327K was present as a minor variant in the index population. The fixation and transmission of NP D53E strongly suggests that this amino acid change is advantageous for efficient replication and transmission in the marmoset host. Although the functional role of this amino acid, located in the PB2-binding domain of NP, is unclear, NP has been implicated in host adaptation in other model organisms [46], [47]. Importantly, the transmission of both NP D53E and PA E327K in the same pair shows that variants present in the index population at both low and high frequencies can be transmitted between hosts.

Together, this study demonstrates that infection with a human 2009pdm isolate results in viral replication, antibody response, symptom development, and transmission in marmoset hosts. Sequence analysis of the infecting viral populations suggests that the species barrier between humans and marmosets may be relatively low, making them a good model for human influenza studies.

## Supporting Information

Figure S1 **Infectious viral titer in BAL and NW fluid from all marmosets.** Infectious viral titer was assessed by standard plaque assay on MDCK cells.
(TIF)

Figure S2 **Infection is not associated with a change in body temperature.** No statistically significant difference in body temperature was observed in infected vs. uninfected animals (unpaired t-test with Welch's correction, p = 0.2398).
(TIF)

Figure S3 **Total protein levels in BAL fluid of all animals.** Total protein levels in BAL fluid of index (blue bars) and contact (green bars) animals were assessed by Bradford assay using a bovine serum albumin (BSA) standard. Levels in infected and uninfected animals are significantly different (unpaired t-test with Welch's correction, p = 0.0002).
(TIF)

Figure S4 **Chemokine levels in BAL fluid of all animals.** Levels of RANTES and MIP-1β in the BAL fluid of infected animals peaks around 10 days post-inoculation/contact. Neither chemokines are observed in the BAL fluid of any uninfected contact animal.
(TIF)

Figure S5 **Average nucleotide diversity across the genomes of viruses isolated from the lower respiratory tract (BAL, panel A) and upper respiratory tract (NW, panel B) of index and contact animals throughout the study.**
(TIF)

File S1 **Table S1, PCR Primer Sequences. Table S2, Reference Genome GenBank Accession Numbers. Table S3, Symptom breakout.**
(DOCX)
