# Supplementary figures and images for: A Novel Nonhuman Primate Model for Influenza Transmission
Source: PLoS One. 2013 Nov 14;8(11):e78750. doi: 10.1371/journal.pone.0078750 (PMC3828296; doi:10.1371/journal.pone.0078750)

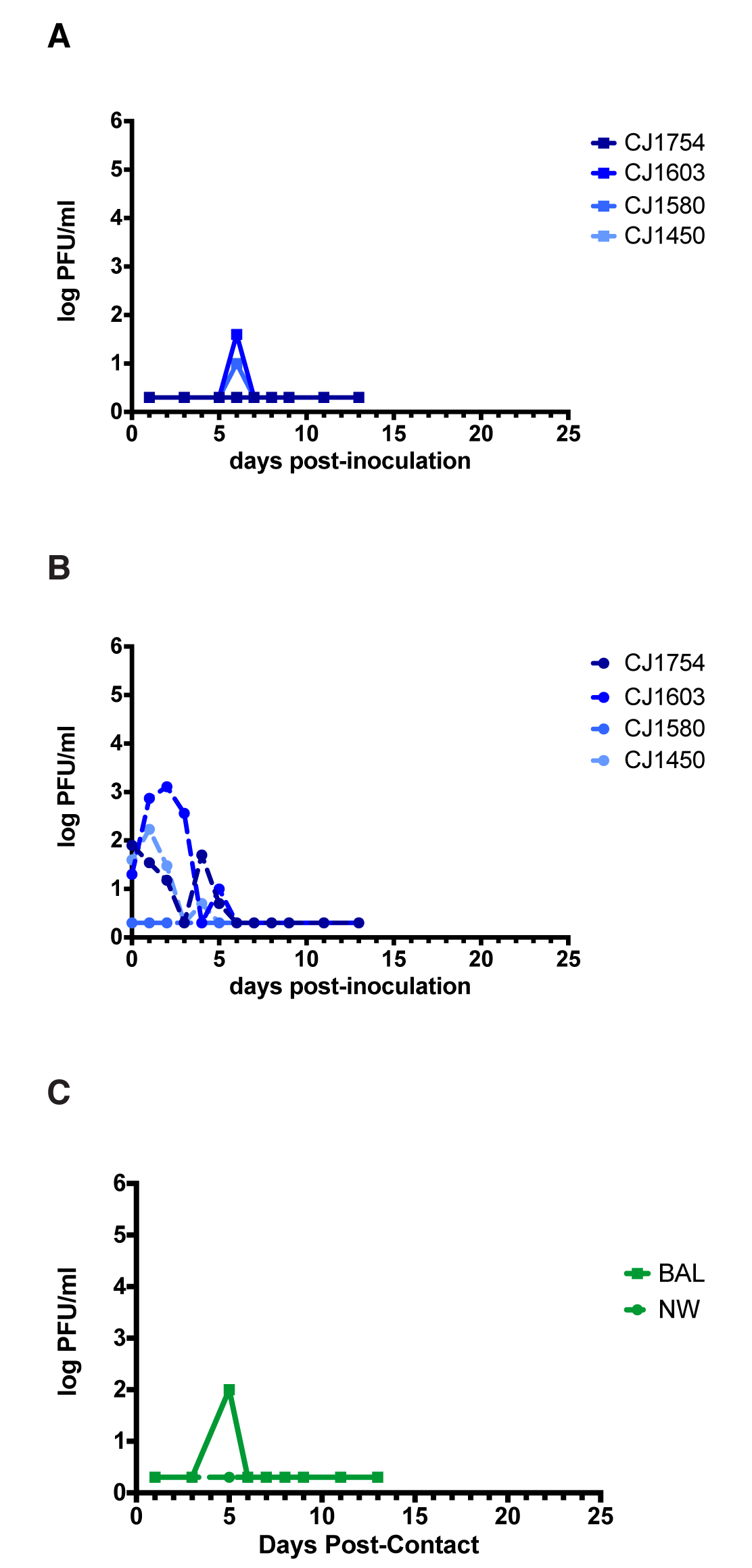

Supplement: Figure S1 — Infectious viral titer in BAL and NW fluid from all marmosets. Infectious viral titer was assessed by standard plaque assay on MDCK cells. (TIF) [file pone.0078750.s001.tif]

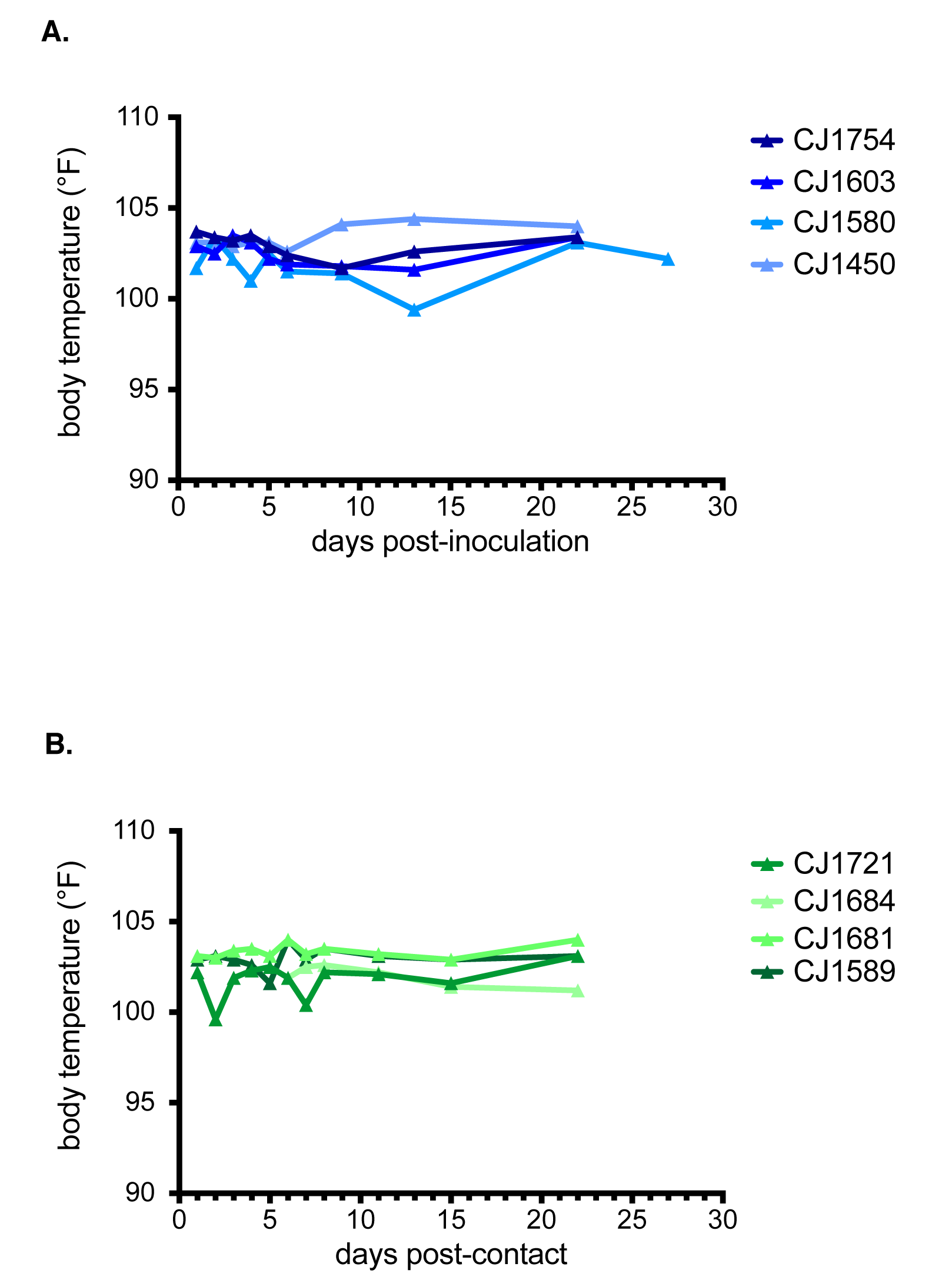

Supplement: Figure S2 — Infection is not associated with a change in body temperature. No statistically significant difference in body temperature was observed in infected vs. uninfected animals (unpaired t-test with Welch's correction, p = 0.2398). (TIF) [file pone.0078750.s002.tif]

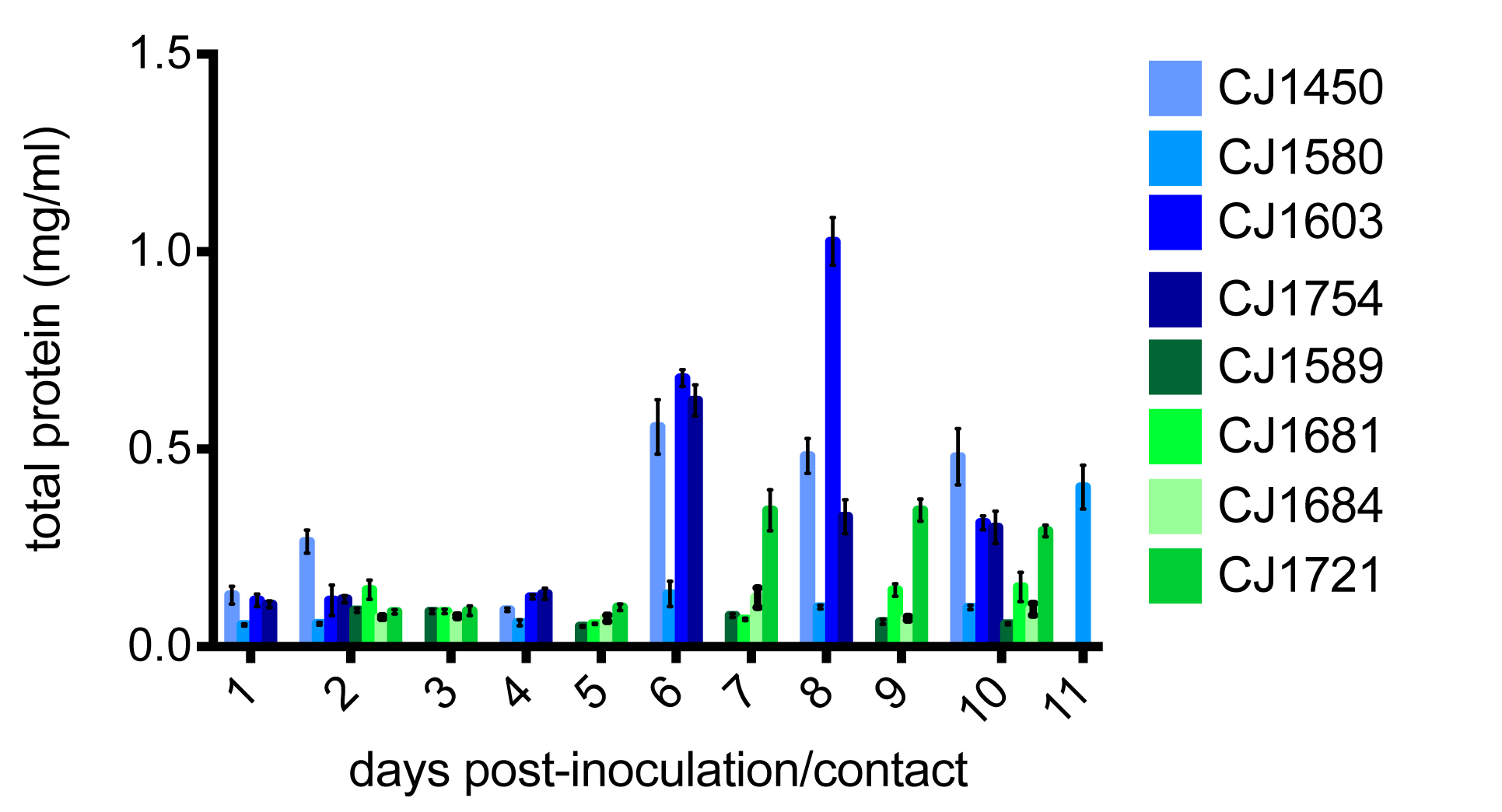

Supplement: Figure S3 — Total protein levels in BAL fluid of all animals. Total protein levels in BAL fluid of index (blue bars) and contact (green bars) animals were assessed by Bradford assay using a bovine serum albumin (BSA) standard. Levels in infected and uninfected animals are significantly different (unpaired t-test with Welch's correction, p = 0.0002). (TIF) [file pone.0078750.s003.tif]

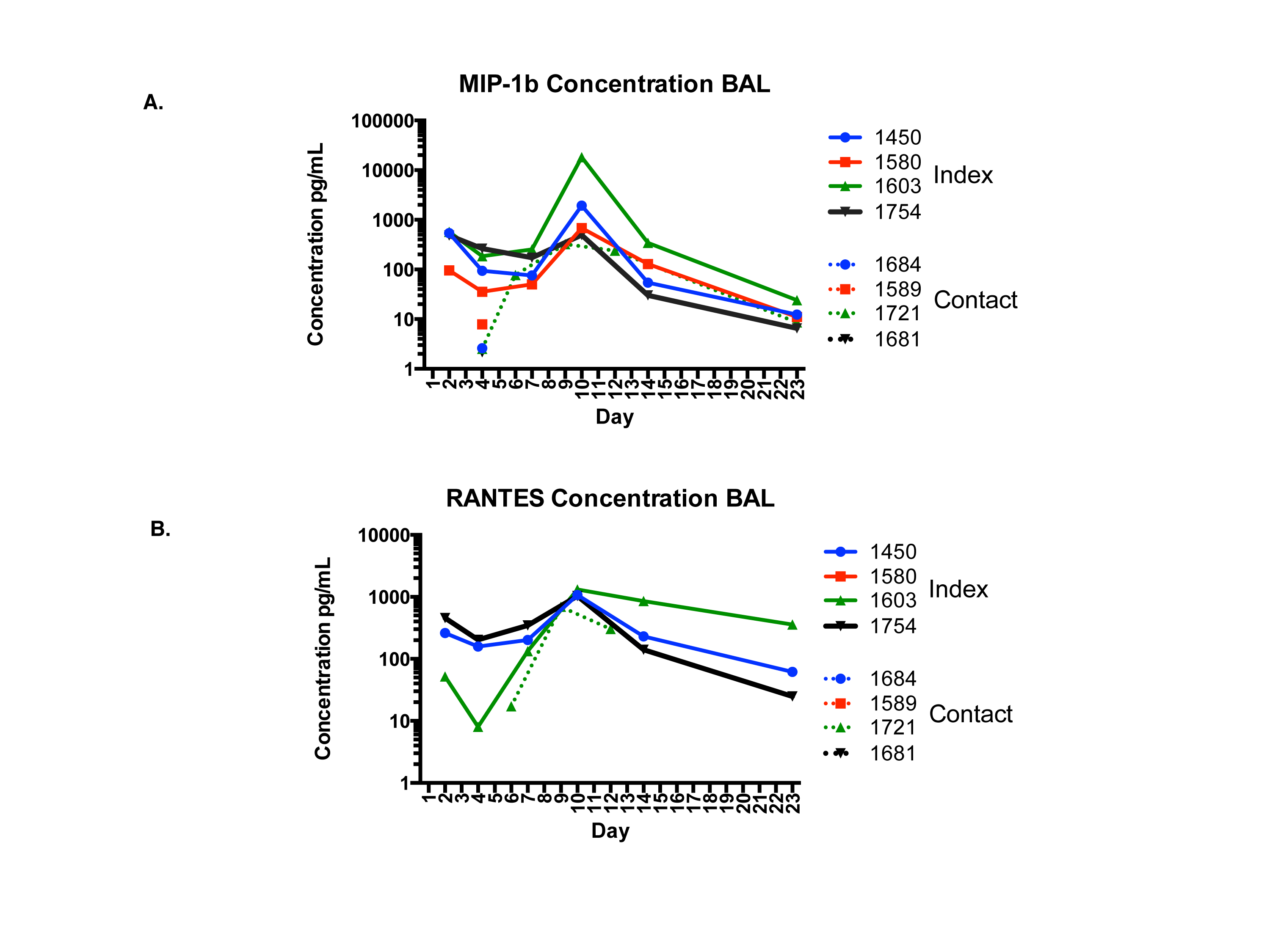

Supplement: Figure S4 — Chemokine levels in BAL fluid of all animals. Levels of RANTES and MIP-1β in the BAL fluid of infected animals peaks around 10 days post-inoculation/contact. Neither chemokines are observed in the BAL fluid of any uninfected contact animal. (TIF) [file pone.0078750.s004.tif]

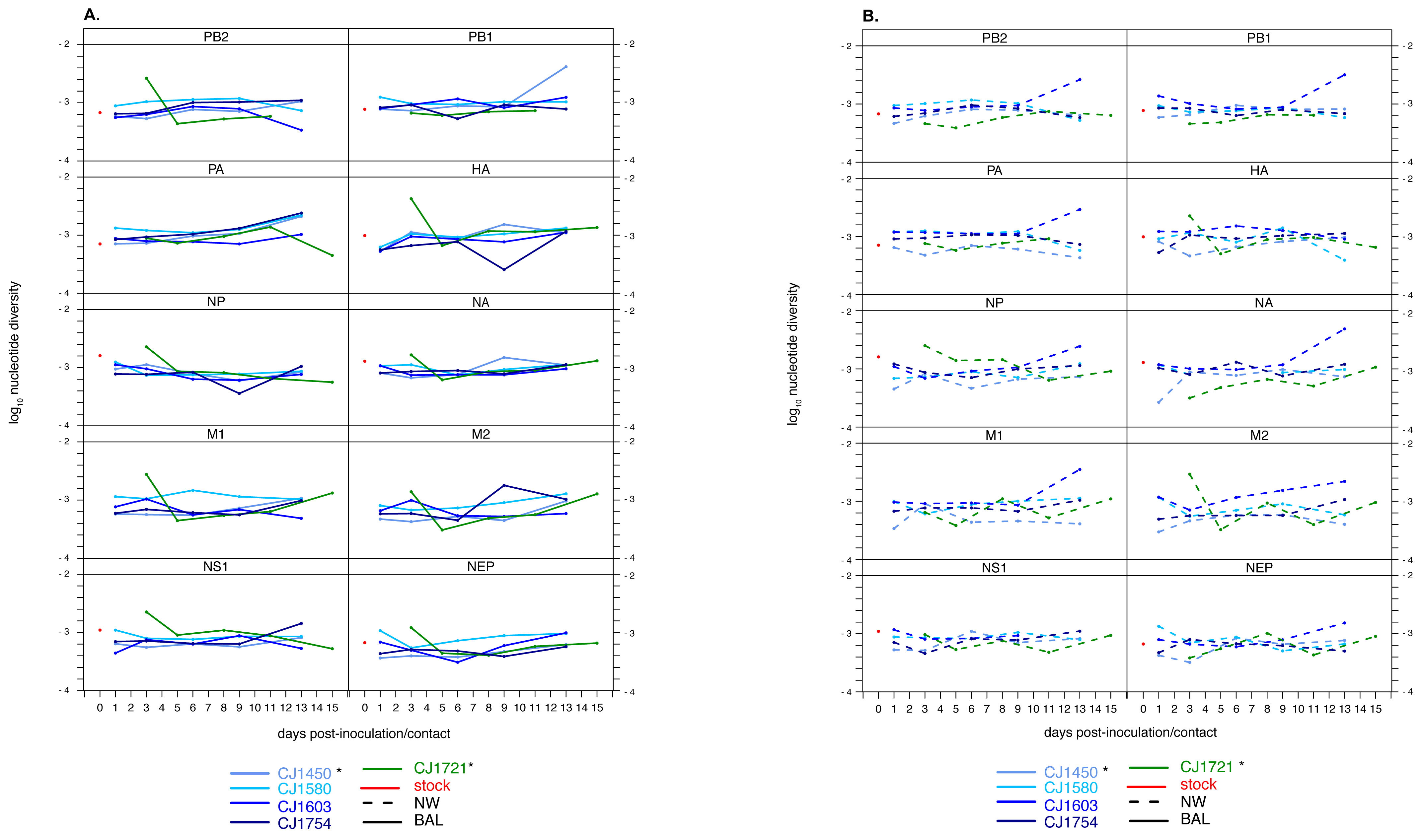

Supplement: Figure S5 — Average nucleotide diversity across the genomes of viruses isolated from the lower respiratory tract (BAL, panel A) and upper respiratory tract (NW, panel B) of index and contact animals throughout the study. (TIF) [file pone.0078750.s005.tif]
