# Supplementary material for: A Novel Nonhuman Primate Model for Influenza Transmission
Source: PLoS One. 2013 Nov 14;8(11):e78750. doi: 10.1371/journal.pone.0078750 (PMC3828296; doi:10.1371/journal.pone.0078750)
Supplement: File S1 — Table S1, PCR Primer Sequences. Table S2, Reference Genome GenBank Accession Numbers. Table S3, Symptom breakout. (DOCX) [file pone.0078750.s006.docx]

## Supplementary Tables

### Table S1: PCR Primer Sequences

| Gene Segment | Forward or Reverse | Primer Sequence |
| --- | --- | --- |
| PB2 | Forward | ATA TAC GCG TAG CGA AAG CAG GTC AA |
|  | Reverse | ATA TAC GCG TAG TAG AAA CAA GGT CG |
| PB1 | Forward | ATA TAC GCG TAG CGA AAG CAG GCA AA |
|  | Reverse | ATA TAC GCG TAG TAG AAA CAA GGC AT |
| PA | Forward | ATA TAC GCG TAG CGA AAG CAG GTA CT |
|  | Reverse | ATA TAC GCG TAG TAG AAA CAA GGT AC |
| HA | Forward | AG CAA AAG CAG GGG AAA ACA A |
|  | Reverse | AG TAG AAA CAA GGG TGT TTT TC |
| NP | Forward | ATA TAC GCG TAG CAA AAG CAG GGT AG |
|  | Reverse | ATA TAC GCG TAG TAG AAA CAA GGG TA |
| NA | Forward | ATA TAC GCG TAG CAA AAG CAG GAT TT |
|  | Reverse | ATA TAC GCG TAG TAG AAA CAA GGA GT |
| M | Forward | ATA TAC GCG TAG CAA AAG CAG GTA GA |
|  | Reverse | ATA TAC GCG TAG TAG AAA CAA GGT AG |
| NS | Forward | ATA TAG GCG TAG CAA AAG CAG GGT GA |
|  | Reverse | ATA TAC GCG TAG TAG AAA CAA GGG TG |

Primer sequences used to amplify viral gene segments.

### Table S2: Reference Genome GenBank Accession Numbers

| Gene Segment | GenBank Accession Number |
| --- | --- |
| PB2 | FJ984387 |
| PB1 | FJ969531 |
| PA | FJ966977 |
| HA | FJ981613 |
| NP | FJ969536 |
| NA | FJ984386 |
| M | FJ966975 |
| NS | FJ969538 |

Genbank accession numbers used for CA/07/09 reference genome.

### Table S3: Symptom breakout

| Days post-inoculation/contact | 1450 | 1580 | 1603 | 1754 | 1721 | 1684 | 1681 | 1589 |
| --- | --- | --- | --- | --- | --- | --- | --- | --- |
| 1 | - | - | - | - | - | - | - | - |
| 3 | - | - | - | - | - | - | - | - |
| 5 | - | - | S | - | - | - | - | - |
| 6 | - | - | - | - | - | - | - | - |
| 7 | - | - | S, ND | - | S | - | - | - |
| 8 | S | S, ND | S, ND, LB | - | - | - | - | - |
| 9 | S | ND | - | S | S | - | - | S |
| 10 | ND | - | S | S, ND | ND | - | - | - |
| 11 | - | S | ND | - | ND | - | - | - |
| 13 | - | - | - | - | - | - | - | - |
| 14 | - | - | S | - | - | - | - | - |
| 15 | - | - | - | S | - | - | - | - |
| 20 | - | - | - | - | S | - | - | - |
| 22 | - | - | - | - | - | - | - | - |
| 27 | - | - | - | - | - | - | - | - |

Instances of symptoms reported for each animal. All infected animals experienced sneezing and nasal discharge on at least 1 day of the study. The most severe symptoms were exhibited by CJ1603, 8 days post-infection, during which sneezing, nasal discharge, and labored breathing were all reported. S = sneezing, ND = nasal discharge, LB = labored breathing, - = no symptoms
